# An arrhythmia classification algorithm using a dedicated wavelet adapted to different subjects

**DOI:** 10.1186/1475-925X-10-56

**Published:** 2011-06-27

**Authors:** Jinkwon Kim, Se Dong Min, Myoungho Lee

**Affiliations:** 1 Department of Electronic and Electrical Engineering, Yonsei University, Seoul, Korea

## Abstract

**Background:**

Numerous studies have been conducted regarding a heartbeat classification algorithm over the past several decades. However, many algorithms have also been studied to acquire robust performance, as biosignals have a large amount of variation among individuals. Various methods have been proposed to reduce the differences coming from personal characteristics, but these expand the differences caused by arrhythmia.

**Methods:**

In this paper, an arrhythmia classification algorithm using a dedicated wavelet adapted to individual subjects is proposed. We reduced the performance variation using dedicated wavelets, as in the ECG morphologies of the subjects. The proposed algorithm utilizes morphological filtering and a continuous wavelet transform with a dedicated wavelet. A principal component analysis and linear discriminant analysis were utilized to compress the morphological data transformed by the dedicated wavelets. An extreme learning machine was used as a classifier in the proposed algorithm.

**Results:**

A performance evaluation was conducted with the MIT-BIH arrhythmia database. The results showed a high sensitivity of 97.51%, specificity of 85.07%, accuracy of 97.94%, and a positive predictive value of 97.26%.

**Conclusions:**

The proposed algorithm achieves better accuracy than other state-of-the-art algorithms with no intrasubject between the training and evaluation datasets. And it significantly reduces the amount of intervention needed by physicians.

## Background

As the healthcare system becomes ubiquitous, the necessity of an automatic diagnosis algorithm increases. In particular, automatic arrhythmia classification algorithm research is the most active area, as arrhythmia is diagnosed by reading long-term data. High-performance arrhythmia classification algorithms based on electrocardiography (ECG) [1-3] have been proposed in many areas over the last several decades. However, the results from these studies have not been applied widely in practice. This situation has arisen due to the differences among the biosignals of different individuals. Particularly, ECG readings from different people show significant differences in terms of their waveform, which can be used for a biometric application [4]. There is no reliable algorithm capable of dealing with these differences thus far.

**Figure 1:**
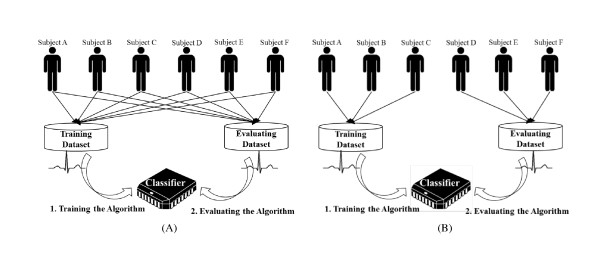
**The configuration of the training and evaluating dataset**. (A) Intrasubject condition, (B) Intersubject condition

Most arrhythmia classification algorithms [1-3] have been evaluated with the same subjects (people) from a training dataset. The results of the aforementioned research showed high performance (more than 95% accuracy), because the algorithm could learn the characteristics of normal and abnormal heartbeats from a subject who was included in both datasets (the training dataset and the evaluation dataset). (We subsequently refer to this assessment condition as the intrasubject condition (Figure 1(a)). However, in practical use, it is impossible to incorporate the ECG data from all humans in a training dataset. Therefore, arrhythmia classification algorithms should analyse ECG data from subjects who are not included in a training dataset.

The intersubject condition is a contrary concept of the intrasubject condition (Figure 1(b)). Superficially, studies undertaken in an intersubject condition demonstrate significantly lower performance levels than those in an intrasubject condition. According to Christov et al. [5], premature ventricular contractions were detected 78.3% in terms of specificity and 81.6% in terms of sensitivity in an intersubject condition, whereas these values were 96.7% in terms of specificity and 96.6% in terms of sensitivity in an intrasubject condition. However, studies in an intersubject condition undergo more appropriate assessments for practical use. To improve the performance in intersubject condition, researchers [6-8] have recently proposed a number of compensation methods. Thus far, these typical compensation methods use a local training dataset.

Chazal et al. [8] evaluated various configurations of feature vectors extracted from ECG data to determine which configuration has robust performance in the intersubject condition. The best performance among the configurations was 75.9% and 77.7% in terms of sensitivity for supraventricular ectopic beat (SVEB) and ventricular ectopic beat (VEB) data, respectively. Chazal and Reilly [7] studied the effect of a local classifier in the intersubject condition to enhance the accuracy of their algorithm. In that study, a physician first verified the subject's data, which was then learned by a local classifier. Their study showed improved performances of 87.7% and 94.3% in terms of sensitivity for SVEB and VEB, respectively. Ince et al. [6] also were able to detect VEB and SVEB respectively at 98.3% and 97.4% in terms of accuracy and 84.6% and 63.5% in terms of sensitivity using a local training dataset. Regarding the use of a local training dataset, other studies [6,7] did not reach a perfect intersubject condition but instead tried to reduce intervention by the physicians by evaluating the effect of various sizes of the local training dataset. In this paper, we propose a new automatic arrhythmia classification method to compensate for the use of the intersubject condition and to improve the performance using a dedicated wavelet.

Wavelets are advantageous because of analyzing a signal in the time-frequency domain using a variety of mother wavelets. Because we can use a variety of mother wavelets, there are already numerous evaluation studies with which to find the optimal wavelet for each application, and this is also true for studies on arrhythmia classification algorithms based on ECG. Using the Haar wavelet, Minhas [9] and Yu [10] achieved high performance by classifying heartbeats with a kth nearest-neighbor (KNN) and a probabilistic neural network (PNN), respectively. Lin proposed an arrhythmia classification scheme which uses the Morlet wavelet as a feature extraction method and a PNN as a classifier [11]. In addition, Froese captured features using the Daubechies 6(db6) wavelet or a wavelet obtained by two proposed wavelet optimization strategies. They compared the performances of various classifiers with the features [12]. In terms of using a deformable wavelet adaptively, such an approach is similar to the proposed algorithm. However, the purpose of their study is to configure the feature vectors by optimal compression of the morphological data, whereas the proposed algorithm has the goal of reducing the differences among the subjects.

In this study, an approach utilizing the continuous wavelet transform (CWT) with a mother wavelet fitted into a target subject's ECG morphology is proposed. Through this process, we expect to reduce the impact of the personal characteristic while obtaining only the differences caused by abnormal heartbeats. The mother wavelet acquired from the signal to process was known as a dedicated wavelet in previous studies [13,14]. The previous studies [13,14] proposed an optimal mother wavelet to perform a voltammetric determination effectively. In this paper, we apply the same method to develop a reliable algorithm to diagnose arrhythmia in an intersubject condition.

## Methods

**Figure 2:**
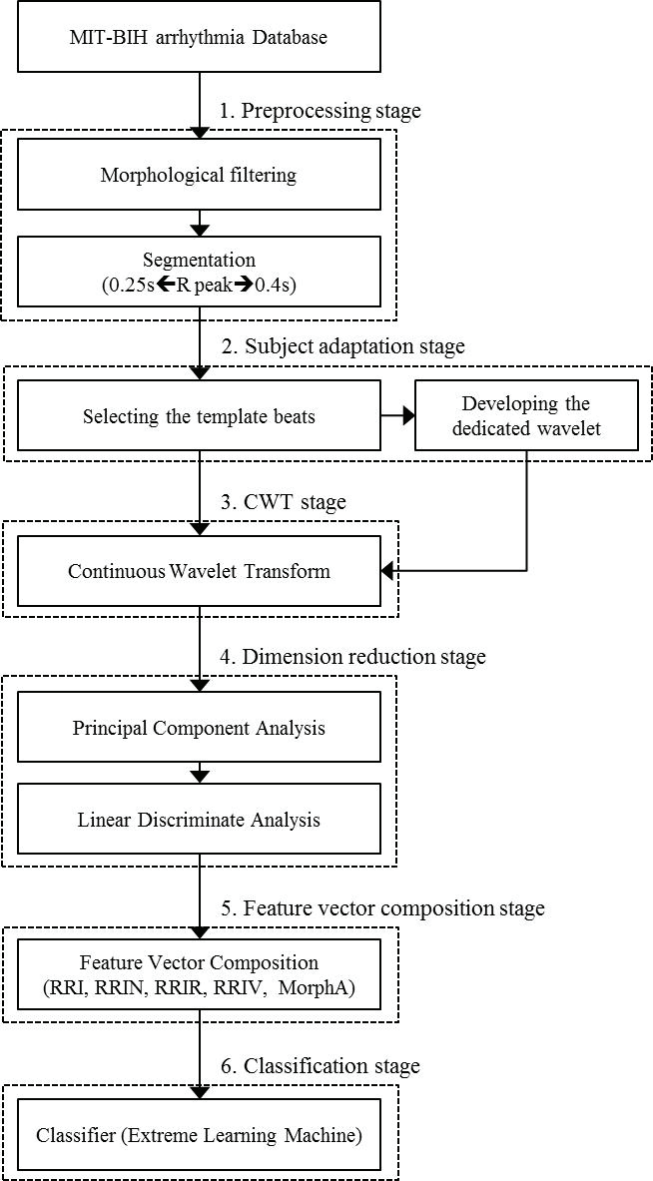
**Block diagram of the proposed algorithm**.

A block diagram of the proposed algorithm is presented in Figure 2. The first required action in an ECG-based arrhythmia classification algorithm is to detect the QRS complex. However, the main purpose of the proposed algorithm focuses on the classification of arrhythmia; consequently, a QRS complex detection process is not included as a part of this research. There are many studies that seek to detect the QRS complex, and these studies have achieved high performance, such as a sensitivity of 99.5% [15,16]. In the proposed algorithm, the annotation data from the MIT-BIH arrhythmia database [17] is used for the implementation and evaluation of the algorithm.

First, the preprocessing step consists of filtering and segmentation. The ECG signal is filtered to remove noise components, and then segmentation is performed to classify each heartbeat. The following subject adaptation stage generates dedicated wavelets using the ECG signal of a target subject. The ECG signal from the target subject is transformed with the dedicated wavelet at the CWT stage. As CWT produces a large amount of transformed data, we utilize principal component analysis (PCA) and linear discriminate analysis (LDA) in the subsequent dimension reduction stage. In the next stage, feature vectors are composed of the compressed morphology data and other features. Lastly, classification is performed.

### Pre-processing

ECG signals are typically exposed to noise components such as power line interference, EMG, respiratory components, and motion artifacts. Therefore, various filtering techniques to remove these noise sources have been proposed. The purpose of filtering techniques is to remove the noise components while preserving the characteristics of the original ECG signal components. In this study, a morphological filtering technique is utilized to remove the noise components. Morphological filtering, which was proposed by Sun [18], has the virtue of removing high-frequency noise and baseline drift with less compromising of the original ECG signal and a low computational burden. When a general filter attenuating a specific frequency band is used, the QRS complex can be expanded because the frequency band of the EMG has some overlap with the band of the QRS complex [2]. This can be a serious weakness in an arrhythmia classification algorithm, because it can increase the likelihood of misclassifying normal heartbeats as VEBs. When using morphological filtering, this phenomenon does not occur, as attenuating specific frequency bands is not used with this type of filtering.

After the morphological filtering process, the ECG signal passes through a segmentation process. Segmentation is performed around the R peak. The PR interval is normally 0.12~0.2 seconds, and T-wave has a duration of about 0.2 to 0.4 seconds. Thus, morphology data 0.25 seconds before the R peak and 0.4 seconds after the R peak are obtained. The major ECG fiducial points (P, T wave and the QRS complex) are included in the segmentation range.

### Subject adaptation method

**Figure 3:**
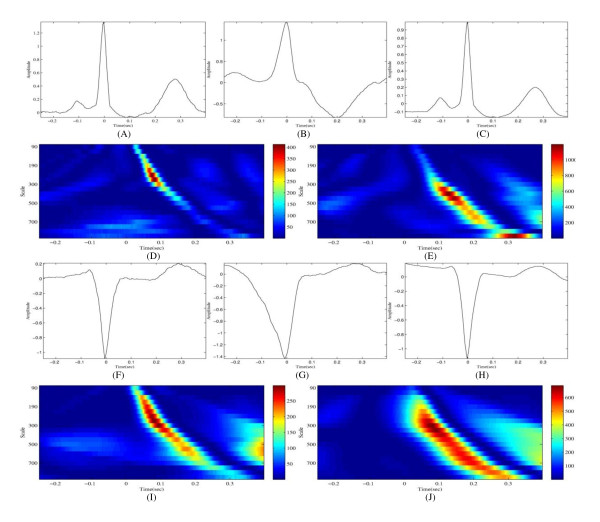
**Examples of the analysis of subjects 106 and 207 in the MIT-BIH arrhythmia database using a dedicated wavelet**. (A) The waveform of subject 106's normal beat, (B) The waveform of subject 106's PVC, (C) The dedicated wavelet of subject 106, (D) The scalogram of subject 106's normal heartbeat using the dedicated wavelet, (E) The scalogram of subject 106's PVC using the dedicated wavelet, (F) The waveform of subject 207's normal beat, (G) The waveform of subject 207's PVC, (H) The dedicated wavelet of subject 207, (I) The scalogram of subject 207's normal heartbeat using the dedicated wavelet, (J) The scalogram of subject 207's PVC using the dedicated wavelet

The template segments are then discerned from each subject to make the mother wavelet similar to the appearance of each subject's normal heartbeat. To reduce noise components, we used an average segment of several consecutive normal heartbeats. In this paper, six consecutive normal heartbeats were used to make template segment. We extracted six consecutive normal heartbeats after passing the first 5 minutes of the ECG record. The mother wavelet is calculated using the template segment. The creation of the optimal mother wavelet is based on Misiti's research [19]. The mother wavelet is generated via least squares approximation. The normal heartbeats and the mother wavelets of subject 106 and 207 are presented below in Figure 3 as an example. The obtained mother wavelets meet the conditions for a square norm of one and a zero mean.

### CWT with a dedicated wavelet

CWT is a type of linear multi-resolution data processing technique that is used to detect or measure the characteristics of signals. CWT analyzes a signal using a scaled and translated version of a mother wavelet to express the signal in the time-scale (frequency) domain. The CWT results are obtained as wavelet coefficients *CWT_x_*(*a,b*), where *a *and *b *refer to the scale and the translation, respectively. *a *is proportional to the inverse of the frequency. *b *represents the location of the wavelet in the time domain. We are able to obtain the information throughout the entire time-frequency plane by varying these values. The CWT coefficients of the input signal *x*(*t*) are as follows.(1)

 in equation (1) indicates the complex conjugation of Ψ_*ab*_(*t*), and Ψ_*ab*_(*t*) is obtained through the process of shifting and scaling the mother wavelet Ψ(*t*), as follows.(2)

As shown in equation (1), each wavelet coefficient denotes the correlation between the signal and the wavelet in the given scale and position. In other words, CWT is used to evaluate how the signal is similar to the scaled and translated mother wavelet. Thus, the optimal mother wavelet in a certain application would have a shape identical to the signal to analyze; we can explain the necessity of a dedicated wavelet based on these ideas.

This study uses sampled data; thus, discrete-time CWT (DT-CWT) is performed. The equations (1) and (2) are represented by equation (3):(3)

*T_s _*in equation (3) refers to the sampling period, it is 2.78 ms in this paper. As shown in equation (3), the value of scale *a *should be determined to obtain the wavelet coefficients of the input signal *x*(**n***T_s_*), and the length of the wavelet coefficients *CWT_x_*(*a*,**k***T_s_*) is identical to that of the input signal at each scale.

The range of scale is chosen to be concentrated in the frequency band of 3~30 Hz, where the main frequency components of the ECG signal exist. The scales approximately relate to the frequency according to equation (4) below.(4)

Here, *F_c _*represents the centre frequency of the mother wavelet, and *F_a _*denotes the pseudo-frequency values corresponding to the scale *a*. The centre frequency of the QRS complex is approximately 5~15 Hz. As shown in Figurer 3, the dedicated wavelets have very similar waveform to each subject's normal heartbeat. Thus, the obtained dedicated wavelets also occupy the same frequency. Table 1 shows *F_a _*values given scale level and the center frequency from 5 to 15 Hz. The highlighted region of Table 1 covers 3~30 Hz of *F_a_*. As shown in Table 1, selected scales from 90 to 1000 covers frequency band 3~30 Hz.

**Table 1:** The frequency value corresponding to each scale level with the centre frequency of the dedicated wavelet

scale	The center frequency of the dedicated wavelet (Hz)					
	
	5	7	9	11	13	15
1000	1.80	2.52	**3.24**	**3.96**	**4.68**	**5.40**
900	2.00	2.80	**3.60**	**4.40**	**5.20**	**6.00**
800	2.25	**3.15**	**4.05**	**4.95**	**5.85**	**6.75**
700	2.57	**3.60**	**4.63**	**5.66**	**6.69**	**7.71**
650	2.77	**3.88**	**4.98**	**6.09**	**7.20**	**8.31**
600	**3.00**	**4.20**	**5.40**	**6.60**	**7.80**	**9.00**
550	**3.27**	**4.58**	**5.89**	**7.20**	**8.51**	**9.82**
500	**3.60**	**5.04**	**6.48**	**7.92**	**9.36**	**10.80**
450	**4.00**	**5.60**	**7.20**	**8.80**	**10.40**	**12.00**
400	**4.50**	**6.30**	**8.10**	**9.90**	**11.70**	**13.50**
350	**5.14**	**7.20**	**9.26**	**11.31**	**13.37**	**15.43**
300	**6.00**	**8.40**	**10.80**	**13.20**	**15.60**	**18.00**
260	**6.92**	**9.69**	**12.46**	**15.23**	**18.00**	**20.77**
230	**7.83**	**10.96**	**14.09**	**17.22**	**20.35**	**23.48**
210	**8.57**	**12.00**	**15.43**	**18.86**	**22.29**	**25.71**
190	**9.47**	**13.26**	**17.05**	**20.84**	**24.63**	**28.42**
170	**10.59**	**14.82**	**19.06**	**23.29**	**27.53**	31.76
150	**12.00**	**16.80**	**21.60**	**26.40**	31.20	36.00
110	**16.36**	**22.91**	**29.45**	36.00	42.55	49.09
90	**20.00**	**28.00**	36.00	44.00	52.00	60.00

The highlighted region covers the range of 3~30 Hz.

CWT transfers a one-dimensional signal to a two-dimensional signal, thus increasing the amount of data considerably. Therefore, a dimension reduction process is adopted to reduce the amount of data in this study.

### Dimension reduction

The output data of CWT is represented by two-dimensional data (images) in the time-scale domain. Each heartbeat is diagnosed based on the obtained morphological features and other features such as the RR interval and the previous heartbeat's classification result. However, because morphological features have a very high dimension, a dimension reduction process is necessary. Linear discriminant analysis (LDA) has been successfully applied to various applications, such as image recognition. However, the small amount of training data with high dimensional features here makes this difficult to apply. As a means of resolving this limitation, LDA after the application of a principal component analysis (PCA) has been widely used [20]. The proposed algorithm also performs PCA and LDA to reduce the dimension of the ECG morphology data transformed by CWT.

The obtained scalogram has 234 samples in the time domain and 20 levels in the scale domain. Thus, 20 × 234 (4680) dimensional morphology features occur for each heartbeat. These are compressed by PCA and LDA to obtain the final four-dimensional feature vectors. The 4680-dimensional dataset was compressed into a 100-dimensional dataset via PCA. At this time, 99.99% of the information based on the Eigenvalues was maintained using the 100-dimensional data. The 100-dimensional dataset was compressed to four dimensions by LDA.

### Feature vector composition

Each heartbeat is classified based on a feature vector configured as shown in Table 2. There are six dimensions of features associated with the RR interval, which are RRI[k], RRIavg, and RRIdiv. RRI[k] represents the features of the RR interval of the second-to-last heartbeat, the last heartbeat, the current heartbeat and the following heartbeat when k equals -2, -1, 0 and 1, respectively. They reflect the information of a premature beat, which normally has a short RR interval and a compensation period. In addition, the average of the RR interval of the last 10 heartbeats is included in the feature vector to establish a baseline. The standard deviation among the last 10 heartbeats is also used to provide information about the variability of the RR intervals at that time.

**Table 2:** The configuration of the feature vector

No	Feature (abbreviation)	Dimension	Description	Equation
1	RRI[k]	4	Adjacent RR intervals	*k *= -2 *t*(*R_peak_*[**n **- 2]) - *t*(*R_peak_*[**n **- 3])
				*k *= -1 *t*(*R_peak_*[**n **- 1]) - *t*(*R_peak_*[**n **- 2])
				*k *= 0 *t*(*R_peak_*[**n**]) - *t*(*R_peak_*[**n **- 1])
				*k *= 1 *t*(*R_peak_*[**n + 1**]) - *t*(*R_peak_*[**n**])
2	RRIavg	1	The average RRI of the last 10 heartbeats	
3	RRIdiv	1	The standard deviation between the RRI of last 10 heartbeats	
4	Annotbf1	5	The classification results of the last heartbeat	[1 0 0 0 0] *if Y*_**n **_= *Normal beat*
				[0 0 1 0 0] *if Y*_**n **_= *SVEB*
				[0 0 1 0 0] *if Y*_**n **_= *VEB*
				[0 0 0 1 0] *if Y*_**n **_= *Fusion beat*
				[0 0 0 0 1] *if Y*_**n **_= *Unknown beat*
5	Annotbf2	5	The classification results of the second-to-last heartbeat	
6	Morph	4	Morphology data of the current heartbeat (After CWT-PCA-LDA)	*LDA*(*PCA*(*CWT*(**X_n_**)))
7	Morphbf1	4	Morphology data of the last heartbeat (After CWT-PCA-LDA)	*LDA*(*PCA*(*CWT*(**X**_**n**-1_)))
8	Morphbf2	4	Morphology data of the second-to-last heartbeat (After CWT-PCA-LDA)	*LDA*(*PCA*(*CWT*(**X**_**n**-2_)))

Physicians diagnose heartbeats using not only information about the current heartbeat but also information about previous heartbeats. Some researchers also used the previous heartbeat's information to classify heartbeats correctly [21]. Therefore, to obtain information about this, Annotbf1 and Annotbf2 are included in the feature vector (features 4 and 5 in Table 2). Annotbf1 refers to the classification result of the last heartbeat, and Annotbf2 refers to the second-to-last heartbeat. These features are acquired from the annotations of the MIT-BIH arrhythmia database in the training phase. However, when evaluating an algorithm, these features come from the results classified by the algorithm at run time. Annotbf1 and Annotbf2 at the beginning of the ECG data are set to normal. Annotbf1 and Annotbf2 are represented in the form of five-dimensional binary features, as shown in Table 2. In addition, Morph, Morphbf1 and Morphbf2 are included (features 6~8 in Table 2) to describe the morphology of the present and the previous heartbeats. Morph, Morphbf1 and Morphbf2 are four-dimensional feature vectors, which are generated by CWT with a dedicated wavelet and PCA-LDA. Morphbf1 and Morphbf2, respectively, represent the morphologies of the last heartbeat and the second-to-last heartbeat. The total dimension of the feature vector is 28. To ensure that the feature vectors are in an appropriate range, each component of the feature vectors, apart from Annotbf1 and Annotbf2, is normalized using equation (5).(5)

*μ_x _*in equation (5) refers to the average of component *x*, and *σ_x _*represents the standard deviation of *x*.

### Classifier

Typically, the Back Propagation Neural Network (BPNN) has some potential disadvantages, such as a slow learning speed, the possibility of convergence to the local minimum and the degradation of the classification performance due to over-training. The Extreme Learning Machine (ELM) proposed by Huang [22] is a new learning technique for multi-layer neural networks which overcomes the limitations of a BPNN. It can obtain the weights of neural network not through iterative learning but through an analytical method. ELM completes the learning process using the Moor-Penrose generalized inverse. Thus, it has the advantage of a high learning speed. In addition, ELM is more convenient to use than a conventional neural network, because only the type of activation function and the number of hidden neurons are necessary to configure the neural network. Several studies have shown that the performance of ELM is higher than that of a BPNN and similar to or slightly lower than that of a support vector machine [2,22]. In this study, ELM is more appropriate than SVM, because of using large training dataset. Therefore, we used ELM due to its fast training speed and high performance. The sigmoid function is used for an activation function, and 50 hidden neurons are used.

### Evaluation Methods

In this study, the MIT-BIH arrhythmia database is used to evaluate the performance of the proposed algorithm. There are five classes of heartbeats according to the AAMI [23]. These are the normal beat class (N class), the supraventricular ectopic beat class (S class), the ventricular ectopic beat class (V class), the fusion beat class (F class), and the unknown beat class (Q class). Matching between the annotations of the MIT-BIH arrhythmia database and the AAMI heartbeat classes is presented in Table 1 of Chazal's paper [8]. The MIT-BIH arrhythmia database is composed of ECG records from 48 subjects with a sampling frequency of 360 Hz, and each record is 30 minutes long. The ECG signals were measured using two leads. Channel A was generally measured by a modified limb lead II (MLII), and channel B by an augmented lead 1 (A1). Some subjects to whom those lead methods were difficult to apply were measured by other lead methods. In this study, only channel A (ML II) is used, and subjects with pacing beats are not included so as to match the AAMI standard. Thus, 44 records were used to configure and evaluate the algorithm after excluding records 102, 104, 107 and 217.

Typically, the morphology signal from the S class is similar to that from the N class. On the other hand, the morphology signal from the V class shows a sizeable difference. Thus, many feature extraction methods for ECG morphology may discriminate the V class from the N class very well in one subject. However, because the morphology from the N class and the V class vary depending on the subject, a normalization process is necessary. The proposed method normalizes the difference in the ECG morphology among subjects using dedicated wavelets. To evaluate the effectiveness of the normalization process, we compared five feature extraction methods. Those are a normal template cross-correlation (TemplateM), a discrete wavelet transform (DWT) with the Haar wavelet (Haar(7)), a DWT with the Daubechies 6 wavelet (db6(4)), a CWT with the dedicated wavelet of subject 106 (106CWT), and a CWT with the dedicated wavelet of each subject (DedicatedW). Each feature extraction method was applied to the ECG signals of the N class and V class, which were extracted from subjects who had over 100 heartbeats of the V class. Using the methods above, we compressed the output data to one-dimensional data via LDA. For 106CWT and DedicatedW, we used the PCA-LDA composition, because these two methods are based on CWT. The compressed data was normalized to remove amplitude variation due to the different signal processing methods.

In terms of using the template segments, TemplateM stands on a basis similar to that of the proposed method. Thus, it is included in this evaluation. Haar(7) and db6(4) are included in this assessment, as these methods are frequently used with many arrhythmia classification algorithms using DWT. Haar(7) and db6(4) indicates that the signal was decomposed up to 7 and 4 levels, respectively. The decomposition levels were set to the extent possible given the support of the signal length. 106CWT is included to evaluate the effect when using the same mother wavelet, similar to the ECG morphology, for all subjects. DedicatedW is the proposed method.

The discrimination ability of the above feature extraction methods was estimated using the Fisher discriminant ratio (FDR). FDR represents how far the data of two classes are spread apart (equation (6)).(6)

In equation (6), *μ_N _*and *μ_V _*denote the data averages from the N class and the V class, respectively, and *σ_N _*and *σ_V _*are the respective standard deviations. The results are represented in Table 3.

**Table 3:** The FDR value for the feature extraction methods

Subject	TemplateM	Haar (7)	db6(4)	106CWT	DedicatedW
106	12.781	**1.098**	5.004	6.409	9.072
116	16.204	3.856	13.851	14.391	30.078
119	30.882	2.279	6.970	11.562	37.488
200	10.937	**1.156**	6.542	5.853	8.136
201	44.911	**0.449**	13.613	13.637	37.534
203	6.146	**0.005**	**0.889**	**0.434**	4.080
207	**1.818**	**0.457**	**1.831**	**0.659**	**1.688**
208	19.283	2.293	15.109	11.629	32.354
210	5.777	**0.780**	3.064	2.289	6.236
213	9.592	**0.772**	5.481	2.986	20.721
214	**1.891**	**0.312**	**1.378**	**1.562**	5.516
215	13.458	**0.088**	**1.162**	**1.488**	6.338
221	21.050	**0.576**	8.558	12.860	33.630
223	**1.085**	**1.155**	2.156	4.963	4.818
228	7.765	**1.372**	5.088	8.809	18.488
233	4.094	**1.645**	3.567	4.624	31.035

Average	12.980	1.143	5.891	6.510	17.951
Std.	11.733	0.997	4.689	4.951	13.604

## Evaluation results and discussion

The scalograms using the dedicated wavelet optimized for each subject (106 or 207) are presented in Figure 3 as an example. The purpose of this study is to reduce the differences from each subject's ECG waveform characteristics while leaving the differences from the types of heartbeats. Figure 3 indicates that this objective is achieved well. Although subjects 106 (top of Figure 3) and 207 (bottom of Figure 3) have very different normal heartbeat waveforms from each other (Figures 3(a) and 3(f)), the normal heartbeat scalograms from these subjects have similar pattern, high components in similar position, through CWT using a dedicated wavelet (Figure 3(c) for subject 106 and (h) for 207). In contrast to the normal heartbeats, the high coefficients of premature ventricular contractions (PVC) are concentrated at the high scale (low frequency, Figure 3(e) and 3(j)).

As shown in Table 3, the FDRs are large when the templates or the mother wavelets are similar to the ECG morphology. (TemplateM, 106CWT and DedicatedW uses the ECG signal as a template or a mother wavelet; some researchers use the db6 wavelet due to its similarity to the ECG morphology [24,25].). Specifically, TemplateM and DedicatedW, which use the ECG of each subject as a template, show the highest performance. We highlight the spaces of Table 3 where FDR is less than 2. The method which has the highest average FDR and the fewest subjects with a small FDR was noted to be DedicatedW. Based on these results, we confirm that DedicatedW has better discrimination ability than any other method.

**Figure 4:**
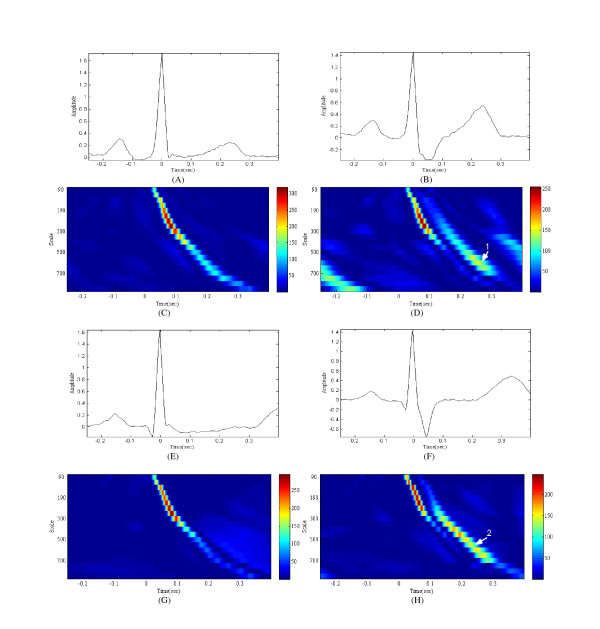
**Examples of the analysis of subjects 212 and 231 in the MIT-BIH arrhythmia database using a dedicated wavelet**. (A) The waveform of subject 212's normal beat, (B) The waveform of subject 212's right bundle branch block(RBBB), (C) The scalogram of subject 212's normal beat using the dedicated wavelet, (D) The scalogram of subject 212's RBBB using the dedicated wavelet, (E) The waveform of subject 231's normal beat, (F) The waveform of subject 231's RBBB, (G) The scalogram of subject 231's normal beat using the dedicated wavelet, (H) The scalogram of subject 231's RBBB using the dedicated wavelet

In addition, while TemplateM can only evaluate the similarity between the template heartbeat and the input signal, the proposed method can analyze other characteristics. There are ECG morphologies and scalograms of a normal heartbeat and the right bundle branch block (RBBB) shown in Feature 4. Both the normal heartbeat and RBBB are included in the N class. However, the morphology of RBBB has a wide and deep S wave owing to its slow right ventricular depolarization (Figures 4(b) and 4(f)).

These differences should be ignored when classifying these two types of heartbeats into the N class. Nonetheless, this is not always feasible when using a method such as TemplateM. The proposed method is able to deal with these types of problems, as it can recognize some characteristics of RBBB through a scalogram. ('1' in Figure 4(d) and '2' in Figure 4(h) both show some low-frequency components when an S wave occurs.)

**Figure 5:**
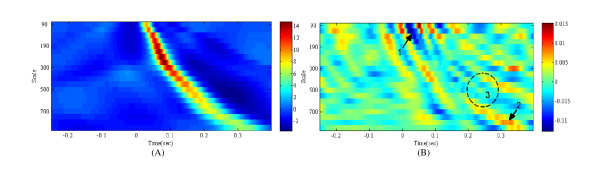
**The average scalogram of the ECG segments (a) and a PCA-LDA mapping matrix (b)**.

Figure 5 shows how the proposed algorithm deals with such a problem. Figure 5 represents the average of the scalograms and the PCA-LDA mapping matrix. The proposed algorithm mapped 20 × 234 (4680) dimensional data to four-dimensional feature vectors through equation (7).(7)

In equation (7), **x**_*in *_and **x**_*out *_are the input and output feature vectors, respectively. **x**_*in *_is a 4680 dimensional data, and **x**_*out *_is a 4 dimensional data. **M**_*PCA*_, **M**_*LDA*_, **μ**_*PCA*_, and **μ**_*LDA *_are the mapping matrix of PCA (4680 × 100), mapping matrix of LDA (100 × 4), mean value of PCA (1 × 4680), and mean value of LDA (1 × 100), respectively [20]. **μ**_*PCA *_is shown in Figure 5(a). As shown in the bottom line of equation (7), only the first term is a variable related to the input feature vectors **x**_*in*_, as the second term and third term are constants. Thus, **M**_*PCA*_·**M**_*LDA *_determine the relationship strength between the input feature vectors and the output feature vectors. Figures 5(b) represents the first component of **M**_*PCA*_·**M**_*LDA*_, which refer to the relationship strength between the first component of the final four-dimensional feature vectors and each coefficients value of the scalogram.

According to the output features through the first PCA-LDA mapping matrix, the features of the V class are generally greater than those of the other classes. As shown in Figure 5(b), the components of the first PCA-LDA mapping matrix are small at a low scale level and near zero in the time domain, where normal scalograms have large components ('1' in (b) in Figure 5). On the other hand, the components are large at a high scale level and 0.2~0.3 in the time domain, where the scalograms of the V class have large components ('2' in (b) in Figure 5). Interestingly, there are virtually zero components in which the scalograms of RBBB have large components ('3' in (b) in Figure 5). Through this process, the proposed method can disregard the unwanted features in the time-frequency domain.

Performance evaluations were carried out in two ways. First, the training and the evaluating datasets were constructed using a leave one out rule to evaluate the algorithm's performance for all of the subjects in the MIT-BIH arrhythmia database. This indicates that the data from 43 subjects were used as the training dataset and that the data from the other one subject was used to evaluate the algorithm. In this case, as the size of the training dataset was too large, with over 100,000 heartbeats, the training dataset was reduced to 15,000 heartbeats (13,300 heartbeats for the N class, 450 heartbeats for the S class, 1200 heartbeats for the V class, 130 heartbeats for the F class and 2 heartbeats for the Q class). The obtained results are presented in Table 4. The proposed algorithm was evaluated in terms of its accuracy, specificity, sensitivity and positive predictive value.(8)(9)(10)(11)

**Table 4:** The results of the proposed algorithm by each subject in the MIT-BIH arrhythmia database

files	beat type					Normal				SVEB				VEB			
	
	N	S	V	F	Q	AC	SF.	ST	+P	AC	SF	ST	+P	AC	SF	ST	+P
100	2239	33	1	0	0	98.50	0	100	98.50	98.55	100	0	-	99.96	100	0	-
101	1860	3	0	0	2	99.68	0	99.95	99.73	99.84	100	0	-	99.95	99.95	-	0
103	2082	2	0	0	0	99.90	0	100	99.90	99.90	100	0	-	100	100	-	-
105	2526	0	41	0	5	97.16	28.26	98.42	98.69	99.49	99.49	-	0	97.67	98.85	24.39	25.64
106	1507	0	520	0	0	99.26	97.31	99.93	99.08	100	100	-	-	99.26	99.93	97.31	99.80
108	1741	4	16	2	0	99.38	68.18	99.77	99.60	99.77	100	0	-	99.26	99.54	68.75	57.89
109	2492	0	38	2	0	98.93	55.00	99.64	99.28	100	100	-	-	99.01	99.64	57.89	70.917
111	2123	0	1	0	0	99.91	100	99.91	100	100	100	-	-	99.91	99.91	100	33.33
112	2537	2	0	0	0	99.92	0	100	99.92	99.92	100	0	-	100	100	-	-
113	1789	6	0	0	0	99.72	16.67	100	99.72	99.67	100	0	-	99.94	99.94	-	0
114	1820	12	43	4	0	97.82	30.51	100	97.80	98.99	99.63	0	0	98.30	100	25.58	100
115	1953	0	0	0	0	100	-	100	100	100	100	-	-	100	100	-	-
116	2302	1	109	0	0	99.67	94.55	99.91	99.74	99.96	100	0	-	99.71	99.91	95.41	98.11
117	1534	1	0	0	0	99.93	0	100	99.93	99.93	100	0	-	100	100	-	-
118	2166	96	16	0	0	95.08	0	100	95.08	95.79	100	0	-	99.90	100	0	-
119	1543	0	444	0	0	100	100	100	100	100	100	-	-	100	100	100	100
121	1861	1	1	0	0	99.95	50.00	100	99.95	99.95	100	0	-	100	100	100	100
122	2476	0	0	0	0	100	-	100	100	100	100	-	-	100	100	-	-
123	1515	0	3	0	0	100	100	100	100	100	100	-	-	100	100	100	100
124	1536	31	47	5	0	97.04	42.17	100	96.97	98.09	100	0	-	99.26	100	74.47	100
200	1743	30	826	2	0	95.69	86.95	100	93.96	98.85	100	0	-	96.92	100	90.31	100
201	1635	128	198	2	0	95.31	73.17	99.76	94.88	93.48	100	0	-	93.17	94.90	77.78	63.11
202	2061	55	19	1	0	98.17	48.00	100	98.14	97.43	100	0		98.64	98.91	68.42	36.11
203	2529	2	444	1	4	97.15	87.80	98.81	97.85	99.93	100	0	-	97.25	98.74	88.74	92.49
205	2571	3	71	11	0	98.42	52.94	99.92	98.47	99.89	100	0	-	98.72	99.81	59.15	89.36
207	1543	107	210	0	0	86.77	23.66	99.74	86.41	94.14	99.89	0	0	92.31	99.70	34.29	93.51
208	1586	2	992	373	2	90.90	80.86	99.56	85.77	99.93	100	0	-	95.47	93.63	99.09	88.72
209	2621	382	1	0	0	90.28	25.07	99.81	90.11	90.31	99.85	24.87	95.96	99.97	99.97	100	50.00
210	2423	22	195	10	0	96.79	62.56	100	96.61	99.17	100	0	-	97.77	99.88	71.28	97.89
212	2748	0	0	0	0	100	-	100	100	100	100	-	-	100	100	-	-
213	2641	28	220	362	0	90.99	51.97	100	90.01	99.14	100	0	-	96.83	99.74	56.82	93.98
214	2002	0	256	1	2	99.20	93.05	100	99.11	100	100	-	-	99.25	99.95	93.75	99.59
215	3196	2	164	1	0	99.49	92.81	99.84	99.63	99.70	99.76	0	0	99.58	99.97	92.07	99.34
219	2082	7	64	1	0	98.93	70.83	99.90	99.00	99.68	100	0	-	99.30	99.90	79.69	96.23
220	1954	94	0	0	0	95.41	0	100	95.41	95.41	100	0	-	100	100	-	-
221	2031	0	396	0	0	99.92	99.49	100	99.90	100	100	-	-	99.92	100	99.49	100
222	2274	209	0	0	0	91.06	43.06	95.47	94.80	91.62	97.01	33.01	50.36	97.74	97.74	-	0
223	2045	73	473	14	0	87.82	43.04	99.95	86.50	97.20	100	0	-	90.98	99.91	50.74	99.17
228	1688	3	362	0	0	99.51	97.81	99.88	99.53	99.85	100	0	-	99.71	99.94	98.62	99.72
230	2255	0	1	0	0	99.96	0	100	99.96	100	100	-	-	99.96	100	0	-
231	1568	1	2	0	0	99.94	66.67	100	99.94	99.94	100	0	-	100	100	100	100
232	398	1382	0	0	0	96.18	97.76	90.70	92.09	96.07	90.70	97.61	97.33	99.89	99.89	-	0
233	2230	7	831	11	0	97.63	91.40	100	96.83	99.77	100	0	-	97.89	99.78	92.78	99.36
234	2700	50	3	0	0	98.18	5.66	100	98.18	98.18	100	0	-	100	100	100	100
Total	90126	2779	7008	803	15	97.18	75.66	99.71	97.21	98.61	99.86	54.44	91.59	98.61	99.56	85.99	93.65

'-' shows the parts with the '0' denominator. The overall performance levels are calculated based on the entire classification results; these do not refer to the average of the performance level of each subject. AC, SF, ST and +P means accuracy, specificity, sensitivity and positive predictive value respectively. SVEB is supraventricular ectopic beats. VEB is ventricular ectopic beats.

In addition, to facilitate a comparison with the findings of Chazal [7,8], the training and evaluating datasets of the second assessment were constructed in the same manner used in those studies. The training dataset (subjects 101, 106, 108, 109, 112, 114, 115, 116, 118, 119, 122, 124, 201, 203, 205, 207, 208, 209, 215, 220, 223 and 230) and the test dataset (subjects 100, 103, 105, 111, 113, 117, 121, 123, 200, 202, 210, 212, 213, 214, 219, 221, 222, 228, 231, 232, 233 and 234) consisted of data from 22 subjects in each case. The details of the possession rate of each class are shown in Table 2 in a referenced study [8]. The size of the training dataset was also too large, with a total of 51,020 beats. Thus, the training dataset was reduced by about 15,000 beats with the same rate. The obtained results are presented in Table 5.

**Table 5:** The classification results of the proposed algorithm by heartbeat type and a comparison with other studies

Proposed algorithm's							**Chazal *et al. *(2006)**[7]						
Real	Results					Total	Real	Results					Total
					
	N	S	V	F	Q			N	S	V	F	Q	
N	44082	115	61	0	0	44258	N	32432	1360	66	483	53	34394
S	302	1448	87	0	0	1837	S	136	1264	31	10	0	1441
V	284	6	2924	7	0	3221	V	45	65	2433	32	4	2579
F	321	0	49	18	0	388	F	24	3	49	216	0	292
Q	5	0	2	0	0	7	Q	4	0	1	0	0	5
Total	44994	1569	3123	25	0	49711	Total	32641	2692	2580	741	57	38711

performance (%): Proposed algorithm							performance (%): Chazal *et al. *(2006)						
	N	S	V	F	Q	Avg		N	S	V	F	Q	Avg

AC	97.81	98.97	99.00	99.24	99.99	97.94	AC	94.39	95.85	99.24	98.45	99.84	94.8
SF	83.28	99.75	99.57	99.99	100	85.07	SF	95.16	96.17	99.59	98.63	99.85	95.52
ST	99.60	78.82	90.78	4.64	0.00	97.51	ST	94.30	87.72	94.34	73.97	0.00	93.89
+P	97.97	92.29	93.63	72.00	0.00	97.26	+P	99.36	46.95	94.30	29.15	0.00	96.53

Chazal *et al. *(2004) [8]							Ince *et al. *(2009) [6]						
					
Real	Results					Total	Real	Results					Total
	N	S	V	F	Q			N	S	V	F	Q	

N	38444	1904	303	3509	98	44258	N	73019	991	513	98	29	74650
S	173	1395	252	16	1	1837	S	686	1568	205	5	6	2470
V	117	321	2504	176	103	3221	V	462	333	4993	79	32	5899
F	33	1	7	347	0	388	F	168	28	48	379	2	625
Q	4	0	3	0	0	7	Q	8	1	3	1	1	14
Total	38771	3621	3069	4048	202	49711	Total	74343	2921	5762	562	70	83658

performance (%): Chazal *et al. *(2004)							performance (%): Ince *et al. *(2009)						
	N	S	V	F	Q	Avg		N	S	V	F	Q	Avg

AC	87.65	94.63	97.42	92.47	99.58	88.58	AC	96.47	97.30	98.00	99.49	99.90	96.62
SF	94.00	95.35	98.78	92.50	99.59	94.35	SF	85.30	98.33	99.01	99.78	99.92	86.76
ST	86.86	75.94	77.74	89.43	0.00	85.88	ST	97.82	63.48	84.64	60.64	7.14	95.58
+P	99.16	38.53	81.59	8.57	0.00	95.06	+P	98.22	53.68	86.65	67.44	0.00	95.84

AC, SF, ST and +P means accuracy, specificity, sensitivity and positive predictive value respectively.

The results of the proposed algorithm show high performance for the N class in all of the subjects. In addition, for the V class, the proposed algorithm shows high performance in most subjects, but some subjects were characterized by low performance. According to some findings [7,8] reporting the performance against each subject, the proposed algorithm shows better performance compared to one study [8] and comparable performance compared to another [7] with the exception of subject 213. The proposed algorithm shows very low ST and +P results for subject 213, as it reports a low ST in the F class and because the subject 213 has many F class heartbeats.

On the other hand, the ST of the S class showed low performance in almost all of the subjects except subject 232. Interestingly, this performance characteristic is common in intersubject research. According to some findings [7,8] as well as the results of the proposed algorithm, those studies show low ST and +P values for the S class in many subjects but achieve high overall performance owing to the high performance only for subject 232. This may be due to characteristics that are too distinctive and the presence of too many S class heartbeats by subject 232. Subject 232 has 1382 S class heartbeats among a total of 2779 S class heartbeats in the entire MIT-BIH arrhythmia database. Moreover, this subject has simultaneously bradycardia and many consecutive atrial premature beats which are in the S class. Therefore, the RRIs of subject 232's normal beats occurred for about 2 seconds, and the RRIs of the atrial premature beats occurred for about 1 second. These characteristics make the feature weak, because the atrial premature beats normally have a RRI of less than 0.6 seconds. This distinctive characteristic of #232 makes the previous heartbeats' information to become important. The proposed algorithm uses information of past heartbeats, such as Annotbf1, Annotbf2, Morphbf1 and Morphbf2, to classify S class heartbeats correctly. Table 6 shows classification results of the proposed algorithm in subject 232 with or without the previous heartbeats' information. Through using the features, the proposed algorithm could classify N and S class more clearly. This kind of previous heartbeats' information, like the number of consecutive S class heartbeats, was also used by other researchers [21].

**Table 6:** The classification results of #232 with or without information of past heartbeats (Annotbf1, Annotbf2, Morphbf1 and Morphbf2)

With information of past heartbeats							Without information of past heartbeats						
Real	Results					Total	Real	Results					Total
					
	N	S	V	F	Q			N	S	V	F	Q	
N	397	1	0	0	0	398	N	398	0	0	0	0	398
S	1	1379	2	0	0	1382	S	864	319	199	0	0	1382
V	0	0	0	0	0	0	V	0	0	0	0	0	0
F	0	0	0	0	0	0	F	0	0	0	0	0	0
Q	0	0	0	0	0	0	Q	0	0	0	0	0	0
Total	398	1380	2	0	0	1780	Total	1262	319	199	0	0	1780

performance (%): Proposed algorithm							performance (%): Chazal *et al. *(2006)						
	N	S	V	F	Q	Avg		N	S	V	F	Q	Avg

AC	99.89	99.78	99.89	100	100	99.80	AC	51.46	40.28	88.82	100	100	42.78
SF	99.93	99.75	99.89	100	100	99.79	SF	37.48	100	88.82	100	100	86.02
ST	99.75	99.78	-	-	-	-	ST	100	23.08	-	-	-	-
+P	99.75	99.93	0.00	-	0.00	-	+P	31.54	100	0.00	-	0.00	-

AC, SF, ST and +P means accuracy, specificity, sensitivity and positive predictive value respectively.

As shown in Table 5, it is very common for existing studies to classify N class heartbeats into the S class. However, the proposed algorithm significantly reduces the number of such misclassifications. This gives the proposed algorithm higher AC, SF and +P values than those of the other studies of the S class. For a V class heartbeat, the proposed algorithm also shows higher performance compared to other research, except for one existing study [7].

On an average performance basis, as obtained by the weighted sum of the performance of each class, the proposed algorithm showed higher AC, ST and +P results, whereas the SF result was slightly lower compared to that of previous studies. This arose because the proposed algorithm misclassified many non-N class heartbeats into the N class. However, in terms of the misclassified heartbeat rate against the entire set of heartbeats, the proposed algorithm misclassified only 2.52% of all heartbeats, while 4.42%, 6.11% and 14.12% of all heartbeats were incorrectly classified in the research of Chazal in 2004 and 2006 and Ince in 2009 [6-8]. In addition, the proposed algorithm is based on 1 channel ECG signal only, but the studies [6-8] used 2 channel ECG signals.

The proposed algorithm specifically uses a different subject-adaptation technique against the existing subject-adaptation algorithms. The aforementioned previous studies [6,7] attempted to adapt the characteristics of a target subject using a part of his data preclassified by a physician in the training dataset. On the other hand, the proposed algorithm does not apply a target subject's data to the training dataset, but uses specific transfer functions to reduce the differences among subjects. In terms of the efforts of physicians, they preclassify 500 heartbeats and then apply them to the training dataset in one previous study [7]. In another [6], they preclassify and apply the first 5 minutes of the data (about 300 heartbeats). The proposed algorithm improved the usability with only 6 heartbeats.

## Conclusions

In this paper, a dedicated wavelet-based arrhythmia classification algorithm is proposed. This algorithm has the characteristic using a mother wavelet optimized for each subject to achieve stable performance, even in the intersubject condition. Through this process, we sought to reduce the variation among the subjects and to preserve only the differences from the arrhythmia. It was verified that this approach works effectively though an assessment of the features and an evaluation of the algorithm. The proposed algorithm is able to ensure higher performance with less effort compared to previous studies. However, the low performance of the S class and V class of some subjects remain as a problem. In addition, the high computational load due to the use of CWT is a disadvantage. We cannot reach a perfect intersubject condition, although those would significantly reduce the amount of intervention needed by physicians. We will continue to develop a robust arrhythmia classification algorithm to deal with these problems.

## Competing interests

The authors declare that they have no competing interests.

## Authors' contributions

JK conceived the study, implemented algorithm and drafted the manuscript. SD participated in the design and coordination of the study, and helped analysis and interpretation of the results. ML finally reviewed the manuscript as corresponding author. All authors read and approved the final manuscript.
